# A case report of diffuse hyperplastic gastropathy with multiple polypoid formations in a patient with pernicious anemia, *Helicobacter pylori* infection, hypergastrinemia and hypoalbuminaemia: Do not forget of Ménétrier’s disease

**DOI:** 10.1016/j.ijscr.2020.11.055

**Published:** 2020-11-12

**Authors:** Daniel Reis Waisberg, Evandro Sobroza de Mello, Francisco Tustumi, Daniel José Szor, Amir Zeide Charruf, Felipe Emanuel Fuhro, Jaques Waisberg, André Roncon Dias

**Affiliations:** a Department of Surgery, Hospital de Clínicas Municipal José Alencar, Sao Bernardo do Campo, Brazil; b Department of Pathology, Instituto do Câncer do Estado de São Paulo (ICESP), Sao Paulo, Brazil; c Digestive and General Surgery Department, ABC Medical School (FMABC), Santo Andre, Brazil

**Keywords:** Hypertrophic gastritis, Gastrectomy, Case report

## Abstract

**Introduction:**

Ménétrier’s disease is a rare condition, frequently associated with *Helicobacter pylori* infection, hypergastrinemia and hypoalbuminaemia.

**Presentation of the case:**

A case of a 55 years-old female patient with a previous diagnosis of pernicious anemia complaining of epigastric discomfort, hyporexia, vomiting, and weight loss is reported. Endoscopy showed multiple gastric polyploid formations and *Helicobacter pylori* infection was detected. Laboratory tests showed elevated gastrin serum levels and presence of antibodies antiparietal cells, as well as microcytic hypochromic anemia compatible with chronic iron deficiency. Albumin serum level was slightly decreased. Full thickness biopsy performed via echoendoscopy reported gastritis cystica/polyposa *profunda*. Given the association of diffuse involvement of the entire stomach, the possibility of developing malignant disease and the clinical symptoms, the patient underwent laparoscopic total gastrectomy with Roux-en-Y reconstruction. The surgical specimen showed the mucosa hyperemic and swollen, with prominent gastric folds. Hyperplastic elongation of gastric foveolas associated with disappearance of oxyntic glands was compatible with Ménétrier’s disease.

**Discussion:**

The Ménétrier’s disease diagnosis may be tricky, especially when an unusual endoscopic presentation is associated with other conditions that may mislead the diagnostic evaluation. The differential diagnoses were gastric malignancies, Zollinger-Ellison syndrome, massive gastric polyposis and gastritis cystica/polyposa *profunda*.

**Conclusion:**

Clinical, laboratory, endoscopic and histopathological findings are paramount for reaching the diagnosis of Ménétrier’s disease, but it should be suspected in all cases of upper gastrointestinal symptoms and hypertrophied gastric mucosa.

## Highlights

• Ménétrier’s disease (MD) is a rare condition. Its incidence remains undetermined.
• MD is frequently associated with *H. pylori* infection, hypergastrinemia and hypoalbuminaemia.
• The gastric mucosal usually presents giant rugal folds with polypoid appearance on upper endoscopy.
• Clinical, laboratory, endoscopic and histopathological findings are paramount for reaching the diagnosis of MD.
• MD should be suspected in all cases of upper gastrointestinal symptoms and hypertrophied gastric mucosa.

## 1 Introduction

Ménétrier’s disease (MD), also known as protein losing hypertrophic gastropathy is a rare condition, whose incidence remains undetermined [1]. The MD’s prognosis is dependent on the main associated conditions, such as *Helicobacter pylori* (HP) infection, hypergastrinemia and hypoalbuminaemia [1]. Rarely, MD is associated with gastric adenocarcinoma, although the exactly risk for malignant transformation is unclear [2].

Epigastric pain with fullness, nausea, vomiting and a generalized peripheral edema secondary to hypoalbuminemia are the most commonly referred symptoms of MD [[1], [2], [3], [4]]. Gastrointestinal bleeding and diarrhea may also be mentioned by the patient [3]. Serum gastrin levels may be elevated due to low gastric acid secretion [4]. Laboratory evaluation most often shows hypoalbuminemia, hypochlorhydria, elevated serum gastrin and iron deficiency anemia [3,4].

In MD, the gastric mucosal usually presents giant rugal folds with polypoid appearance on upper endoscopy, which are considered the hallmark of the disease [3]. The diagnosis depends on the full-thickness biopsy, showing the loss of the deep glandular component [4]. However, the diagnosis may be tricky, especially when an unusual endoscopic presentation is associated with other conditions that may mislead the diagnostic evaluation. There is no evidence-based guideline for MD treatment. It initially consists of proton-pump inhibitors, high-protein diet, octreotide long-acting release and occasionally intravenous albumin infusion [1,4]. Cetuximab, a monoclonal antibody against EGF (epidermal growth factor), has been associated with regression of disease [1]. Nevertheless, surgical resection still remains the only definite treatment for severe causes with intractable symptoms and massive protein loss [1,3].

We herein present a diagnostic challenging Ménétrier’s disease case report in a patient with a previous diagnosis of pernicious anemia and discuss how the diagnosis rationale was made. This manuscript has been reported in line with the SCARE criteria [5].

## 2 Case summary

**Fig. 1:**
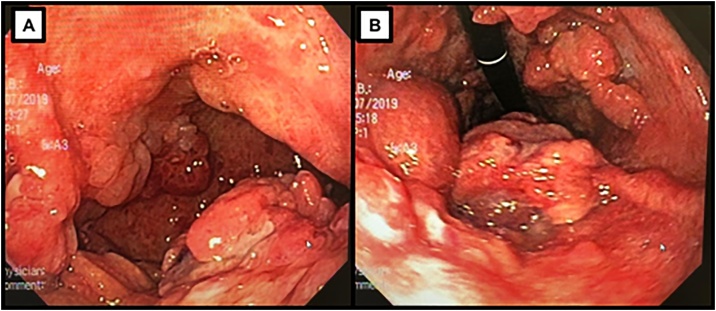
Upper endoscopy showing swollen, granular and hyperemic mucosae with multiple polyploid formations (A), which were pearly and friable and occasionally presented superficial erosions (B).

A 55 years old female complained of epigastric discomfort, hyporexia, vomiting, and weight loss. Endoscopy showed multiple pearly and friable gastric polyploid formations with superficial erosions and active blood oozing (Fig. 1). The stomach presented reduced distensibility and the mucosae was swollen, granular and hyperemic. Sample biopsies showed hyperplasic gastric polyps with HP infection.

Her past medical history included hypothyroidism and previous diagnosis of pernicious anemia treated with quarterly intramuscular vitamin B12 reposition. She also presented iron deficiency (20.4 μg/dL) and received intravenous iron reposition on regular basis. Her mother and a maternal uncle died of rectum cancer and gastric cancer when they were 52 and 60 years, respectively. Physical examination was unremarkable. Laboratory test showed elevated serum gastrin (694 pg/dL), presence of antiparietal cells antibodies (1/80) and microcytic hypochromic anemia (Hemoglobin 10.9 g/dL, Hematocrit 38.8 %, mean globular volume 75 mcm^3^, mean globular hemoglobin 21 pg). Albumin serum levels were slightly decreased (3.22 mg/dL) and B12 vitamin levels were normal (479 pg/mL).

**Fig. 2:**
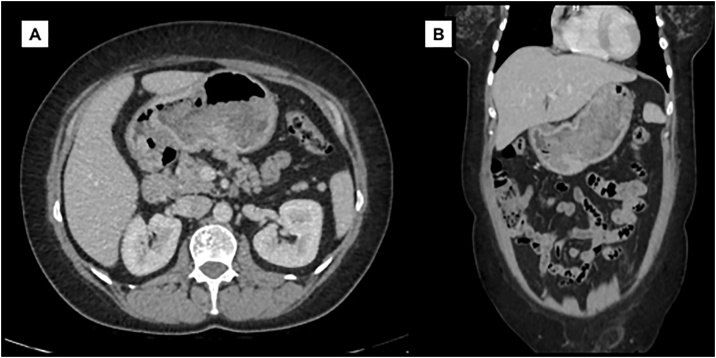
Abdominal computed tomography presenting diffuse gastric mucosa enhancement with no signs of extramural extension or abdominal lymphomegalies. The stomach appears bulky and filled with protruding luminal content from the mucosae (A, axial view; B, coronal view).

Abdominal computed tomography presented diffuse gastric mucosa enhancement with no signs of extramural extension or abdominal lymphomegalies (Fig. 2). Colonoscopy was normal. Echoendoscopy exhibited atrophic and irregular mucosae with multiple and large polyploid lesions, without involvement of the muscularis propria layer. Full thickness biopsy reported gastritis cystica/polyposa *profunda* (GCPP).

Given the combination of chronic bleeding, weight loss, the possibility of developing malignant disease and the diffuse involvement of the entire stomach, the patient underwent laparoscopic total gastrectomy with Roux-en-Y reconstruction. She was treated for HP infection prior to surgery. The stomach was large and bulky making its mobilization difficult. Postoperative evolution was uneventful and she was discharged home on post-operative day 10. The procedure was performed by a senior gastrointestinal surgeon with vast experience in gastric cancer surgery, who is a staff surgeon in a large oncologic center in Brazil with high-volume of gastrectomies due to malignant causes.

**Fig. 3:**
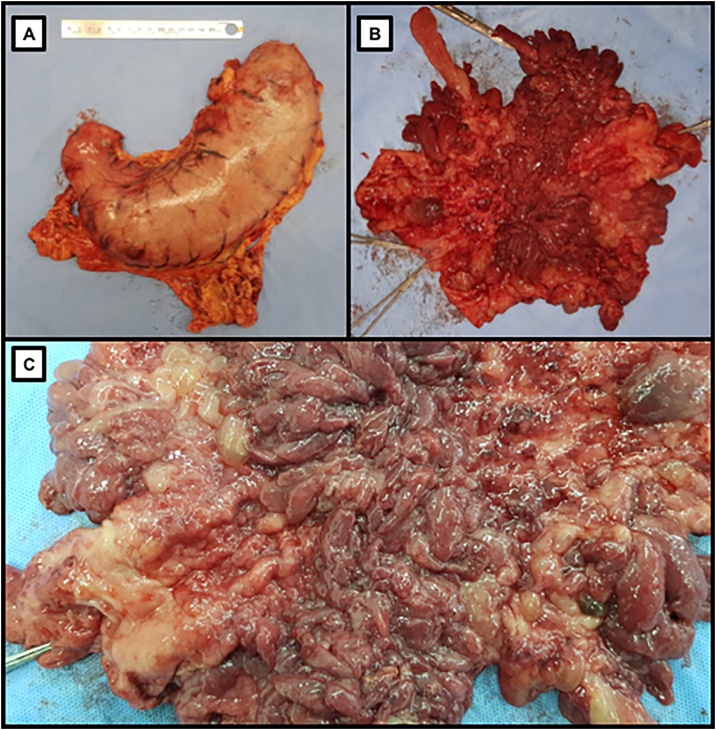
Resected stomach with regular external aspect (A). Specimen cut along the greater curvature, revealing important and diffuse enlargement of gastric folds, with friable polypoid formations of varying sizes and deposition of thick mucus in some areas of the mucous surface (B). Closer view of the polypoid formations (C).

**Fig. 4:**
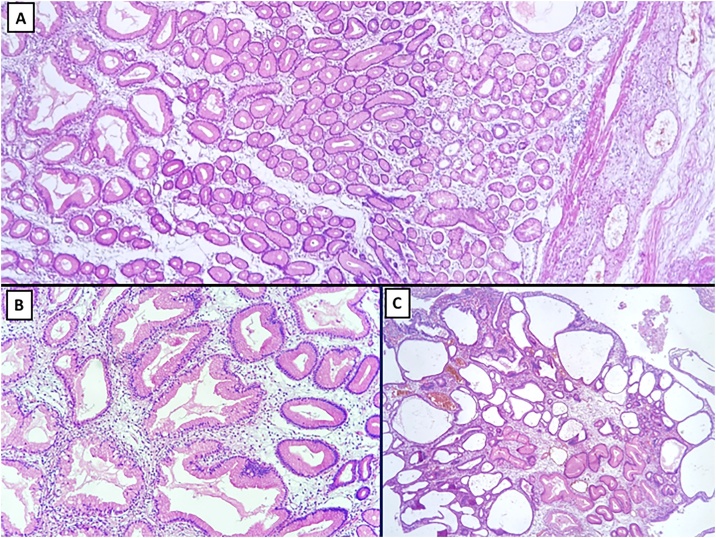
Low power view of gastric mucosa with marked elongation and tortuosity of foveolae and disappearance of oxyntic glands. Submucosa has no glands at all (A). A closer view of mucous cell (foveolar) hyperplasia (B). Polypoid lesions had hyperplastic foveolae that are superficially dilated (C).

On gross examination, the surgical specimen showed the mucosa hyperemic and swollen, with prominent gastric folds and multiple friable polypoid formations in the entire stomach (Fig. 3). Histopathological analysis revealed diffuse and marked hyperplastic elongation of gastric foveolas associated with disappearance of oxyntic glands, compatible with MD (Fig. 4). Besides hyperplasia, polypoid formations frequently disclosed cystic dilation of foveolae without any submucosal involvement. No evidence of neoplasia was found on 16 lymph nodes. The patient remains well in outpatient follow-up 6 months after surgery. Interestingly, albumin, hemoglobin and iron serum levels have increased to 4.61 g/dL, 12.6 g/dL and 104 μg/dL, respectively.

## 3 Discussion

We presented a case of Ménétrier’s Disease (MD) only diagnosed after the patient underwent total gastrectomy. The preoperative diagnosis was not made because of the unusual presentation on upper endoscopy - which did not report giant rugal edematous enlarged gastric folds - associated with a misleading histopathological diagnosis of GCPP. The patient also had pernicious anemia, which leads to hypergastrinemia and hypochlorhydria, further hindering the diagnosis. Retrospectively analyzing the case, the clinical symptoms, iron deficiency anemia and hypoalbuminemia could be explained by MD, which is also strongly associated with HP infection [4].

At low power, a biopsy of MD may appear similar to a hyperplastic polyp, with elongated, tortuous and sometimes cystically dilated gastric foveolae. The diffuse nature of mucous cells (foveolar) hyperplasia in MD makes the distinction. A few other diseases present with macroscopically enlarged mucosal folds in the stomach, making the diagnosis of MD occasionally challenging. For instance, besides gastric malignancies, Zollinger-Ellison syndrome (ZES), massive gastric polyposis (MGP) and GCPP are the main differential diagnosis [6,7]. The presence of parietal cell hyperplasia instead foveolar hyperplasia would characterize ZES. MGP is a designation often applied to severe gastric involvement by juvenile polyposis, with microscopically very distinctive hamartomatous polyps. Finally, GCPP is an uncommon pseudotumoral condition characterized by hyperplasia of foveolar epithelium with cystically dilated glands immersed in submucosa, an absent finding in our case. It is commonly associated with previous gastroenteroanastomoses, although it may be found in unoperated stomach [8]. Most of the time, it is observed as a mass protruding towards the gastric cavity, a pattern distinct from the diffuse involvement found in the present case.

The patient was successfully treated with laparoscopic total gastrectomy. Other less invasive therapies for MD, such as proton-pump inhibitors, high-protein diet, octreotide long-acting release and cetuximab [4], could not be attempted given the diagnosis was postoperative. Nevertheless, their probability of success was low due to the massive involvement of the disease. Surgical treatment is still recommended for cases with persistent symptoms or when there is concern for gastric malignancy [9,10].

Pereyra et al. [11] also reported a case of MD diagnosed postoperatively. The indication for partial gastrectomy was due to an invasive adenocarcinoma diagnosed in the stomach. There is a known association between MD and cancer, probably due to a multiple factors, such as mucosal atrophy, changing in the luminal pH and bacterial colonization, and production of nitrosamines [2].

Even though Menetrier’s disease usually affects mainly the fundus and body of the stomach, total gastrectomy has been preferred over partial gastrectomy to avoid the risk for symptoms persistence, and the risk for malignant transformation in the gastric remnant along lifetime [9,10,[12], [13], [14]]. For gastric cancer, the risk for postoperative severe complications seems to be higher in total gastrectomy [14] and quality of life [15] seems to be poorer in total gastrectomy. However, no studies comparing total and partial gastrectomy for MD have ever been performed. Patients and surgeons should be aware of these issues when deciding which surgical resection would be performed. In our case, the entire stomach was compromised, thereby total gastrectomy was warranted. Laparoscopic and robotic gastrectomy has been described for MD and is feasible [4,16], offering the benefits of a minimally invasive procedure. As MD is a benign disease, there is no need of extensive lymphadenectomy, which makes the procedure technically less demanding when compared with gastrectomy for advanced gastric adenocarcinoma. However, it should be noted that the stomach in cases of MD is often large and bulky, adding difficulty to its mobilization during minimally invasive gastrectomy.

In conclusion, clinical, laboratory, endoscopic and histopathological findings are paramount for reaching the diagnosis of MD, but it should be suspected in all cases of upper gastrointestinal symptoms and hypertrophied gastric mucosa.

## Declaration of Competing Interest

The authors report no declarations of interest.

## Funding

The authors received no specific funding for this work.

## Ethical approval

Ethical approval exemption was given for this study.

## Consent

Written informed consent was obtained from the patient for publication of this case report and accompanying images. A copy of the written consent is available for review by the Editor-in-Chief of this journal on request.

## Registration of research studies

A case report of diffuse hyperplastic gastropathy with multiple polypoid formations in a patient with pernicious anemia, *Helicobacter pylori* infection, hypergastrinemia and hypoalbuminaemia: do not forget of Ménétrier’s disease.

UIN6125

https://www.researchregistry.com/browse-the-registry#home/.

## Guarantor

Francisco Tustumi.

## Provenance and peer review

Not commissioned, externally peer-reviewed.

## CRediT authorship contribution statement

**Daniel Reis Waisberg:** Writing - original draft. **Evandro Sobroza de Mello:** Writing - review & editing, Formal analysis, Investigation. **Francisco Tustumi:** Methodology. **Daniel José Szor:** Writing - original draft. **Amir Zeide Charruf:** Writing - review & editing, Formal analysis, Investigation. **Felipe Emanuel Fuhro:** Validation, Supervision. **Jaques Waisberg:** Conceptualization. **André Roncon Dias:** Conceptualization.

## References

[1] Lambrecht N.W. (2011). Ménétrier’s disease of the stomach: a clinical challenge. Curr. Gastroenterol. Rep..

[2] Remes-Troche J.M., Zapata-Colindres J.C., Starkman I., De Anda J., Arista-Nasr J., Valdovinos-Diaz M.A. (2009). Early gastric cancer in Menetrier’s disease. Case Rep..

[3] Azer M., Sultan A., Zalata K., Abd El-Haleem I., Hassan A., El-Ebeidy G. (2015). A case of Menetrier’s disease without Helicobacter pylori or hypoalbuminemia. Int. J. Surg. Case Rep..

[4] Parianos C., Aggeli C., Sourla A., Zografos G.N. (2020). Total gastrectomy for the treatment of Menetrier’s disease persistent to medical therapy: a case report. Int. J. Surg. Case Rep..

[5] Agha R.A., Borrelli M.R., Farwana R., Koshy K., Fowler A., Orgill D.P., SCARE Group (2018). The SCARE 2018 statement: updating consensus Surgical CAse REport (SCARE) guidelines. Int. J. Surg..

[6] Trinh V.Q., Shi C., Ma C. (2020). Gastric neuroendocrine tumors from long-term proton pump inhibitor users are indolent tumors with good prognosis. Histopathology.

[7] Machicado J., Shroff J., Quesada A. (2014). Gastritis cystica profunda: endoscopic ultrasound findings and review of the literature. Endosc. Ultrasound.

[8] Ramrakhiani N.S., Shetler S.A., Lombard C., Triadafilopoulos G. (2018). Gastritis cystica polyposa: a rare cause of abdominal pain and early satiety treated with endoscopic resection. Dig. Dis. Sci..

[9] Norero E., Vargas C., Achurra P. (2019). Survival and perioperative morbidity of totally laparoscopic versus open gastrectomy for early gastric cancer: analysis from a single latin american centre. Arq. Bras. Cir. Dig..

[10] Pryczynicz A., Bandurski R., Guzińska-Ustymowicz K., Niewiarowska K., Kemona A., Kędra B. (2014). Ménétrier’s disease, a premalignant condition, with coexisting advanced gastric cancer: a case report and review of the literature. Oncol. Lett..

[11] Pereyra L., Gómez E.J., Mella J.M., Casas G., Bugari G., Cimmino D., Pedreira S., Boerr L.A. (2011). Diffuse gastric cancer associated with Ménétrier’s disease. Acta Gastroenterol. Latinoam..

[12] Brownson E., Stanley A.J., Konanahalli P., Seenan J.P. (2019). Unusual case of adult familial Menetrier disease in siblings. BMJ Case Rep. CP.

[13] Byun J., Kwon S., Oh S.Y., Lee K.G., Suh Y.S., Kong S.H., Kim S.G., Kim W.H., Yang H.K., Lee H.J. (2014). Laparoscopic management of hypertrophic hypersecretory gastropathy with protein loss: a case report. Asian J. Endosc. Surg..

[14] Papenfuss W.A., Kukar M., Oxenberg J., Attwood K., Nurkin S., Malhotra U., Wilkinson N.W. (2014). Morbidity and mortality associated with gastrectomy for gastric cancer. Ann. Surg. Oncol..

[15] Hjermstad M.J., Hollender A., Warloe T., Otto Karlsen K., Ikonomo I., Kvaloy S., Nome O., Holte H. (2006). Quality of life after total or partial gastrectomy for primary gastric lymphoma. Acta Oncol..

[16] Rodríguez Gonzalez O., José R., Génesis J., Luis M., Liumariel V., Raquel F., Alexis S. (2015). Robot-assisted laparoscopic gastrectomy for Menetrier’s disease. J. Robot. Surg..

